# Optimizing the Indoor Air Quality in Historical Buildings: Strategies for Environmental Improvement and Public Health Enhancement

**DOI:** 10.3390/ijerph21030341

**Published:** 2024-03-14

**Authors:** Prisco Piscitelli, Alessandro Miani, Saverio Mecca, Rachel Hodgton

**Affiliations:** 1 Italian Society of Environmental Medicine (SIMA), Viale di Porta Vercellina, 9, 20123 Milan, Italy; saverio.mecca@gmail.com; 2 Department of Experimental Medicine, University of Salento, Via Monteroni, 73100 Lecce, Italy; 3 International WELL Building Institute, New York, NY 10001, USA; rachel.hodgdon@wellcertified.com

The endeavor to maintain and enhance the indoor air quality (IAQ) in historical buildings transcends the traditional boundaries of cultural heritage preservation, emerging as a pivotal public health concern. These structures, which stand as testaments to the rich and diverse tapestry of human history, are often beleaguered by air quality challenges, which can pose substantial health risks to both visitors and staff. The intricate nature of these structures, coupled with their age and the materials used in their construction, often results in a unique set of IAQ issues. These range from the accumulation of dust and particulate matter to the presence of volatile organic compounds (VOCs) and other pollutants, which can emanate from the building materials themselves, artifacts housed, and visitors [1].

Furthermore, the presence of other pollutants such as mold, dust, and chemical vapors as a result of conservation practices can create an environment detrimental to human health. The research [2,3,4,5,6,7,8,9,10,11] has shown that prolonged exposure to poor IAQ can lead to a range of health problems, including respiratory issues, allergies, and even more severe chronic conditions. This is particularly concerning in historical buildings, which are not only workplaces but also popular tourist destinations, drawing large numbers of visitors who may be unaware of their potential air quality hazards. The challenge is to address IAQ issues at historical sites without compromising their integrity.

Protecting the health of individuals working in historical buildings is an essential aspect of enhancing their IAQ. These individuals, including curators, conservationists, conservation scientists, scholars, administrative staff, and maintenance crews, spend significant amounts of time within these environments, making them particularly susceptible to the long-term effects of poor air quality. Prolonged exposure to pollutants such as dust, mold spores, chemical vapors from conservation materials, and even potentially harmful emissions from aging building materials can lead to chronic respiratory issues, allergies, and other health concerns [5,6,12].

From an environmental chemistry perspective, the first step in safeguarding the health of workers in historical buildings involves a comprehensive assessment of the air quality. This includes identifying and quantifying the various pollutants present, such as VOCs, particulate matter, and biological contaminants like mold, using advanced monitoring techniques. Equally, advanced analytical techniques, like gas chromatography–mass spectrometry (GC-MS) and high-performance liquid chromatography (HPLC), can be employed to accurately detect and measure pollutant levels. Once they are identified, the source of these pollutants must be addressed. 

For instance, if certain building materials or preservation chemicals are significant sources of VOCs, alternatives with lower emission rates must be sought out. In terms of environmental medicine, the focus shifts to understanding the health implications of these pollutants and implementing strategies to mitigate their impact. Regular health screenings and monitoring for staff working in these environments can help with the early detection of potential health issues. Additionally, educating staff about the symptoms of exposure to various pollutants and the importance of protective measures is crucial. This could include training on the proper use of personal protective equipment (PPE), such as masks and gloves, especially during conservation processes, which might involve the release of harmful substances.

Innovative technologies also play a pivotal role in sanitizing indoor environments in historical buildings. One such technology is the use of advanced HVAC systems with HEPA filters and UV-C light sanitization [13,14,15,16]. HEPA filters are highly effective in trapping particulate matter, including mold spores and dust, while UV-C light has been proven to inactivate a wide range of microorganisms, thus reducing the biological load in the air. Furthermore, the integration of smart sensors and IoT (Internet of Things) [17,18] technology with new AI (Artificial Intelligence) and Neural Network algorithms [19] can enable real-time monitoring of the IAQ, allowing for prompt responses to any deterioration in air quality.

Another innovative approach is the use of photocatalytic and electrocatalytic oxidation (PCO and ECO) technologies [20,21,22]. With these technologies, a photocatalyst, typically titanium dioxide, is utilized to oxidize organic pollutants in the air, converting them into harmless substances like water and carbon dioxide. PCO systems can be particularly effective in reducing the concentration of VOCs and other organic compounds in the air.

Additionally, the implementation of green cleaning practices in historical buildings can significantly reduce the introduction of new pollutants. This involves using cleaning products that are environmentally friendly and free from harsh chemicals, which may contribute to poor IAQ. Moreover, adopting practices like controlled ventilation during cleaning and conservation processes can help by minimizing the concentration of pollutants in the air.

In addition to technical and health-focused strategies, it is imperative for policymakers, governments, and international organizations such as UNESCO, ICOM, ICCROM, and the European Union to shape and enforce policies that prioritize indoor air quality in historical buildings [23,24]. These entities should collaborate to establish comprehensive guidelines and standards for IAQ management at cultural heritage sites. This includes setting permissible levels for various pollutants, mandating regular air quality assessments, and enforcing the use of environmentally friendly materials and practices in conservation efforts.

Funding and resources are crucial to this endeavor. Governments and international bodies should allocate specific funds dedicated to improving the IAQ in historical buildings. This financial support could be used for upgrading HVAC systems, implementing advanced air purification technologies, and conducting essential research on environmental chemistry and medicine pertaining to IAQ. Furthermore, incentivizing research and development in this field can lead to the innovation of more effective and sustainable solutions.

The relevant policy frameworks should also include educational and training programs for staff working in these buildings, ensuring they are well informed about IAQ issues and mitigation measures. Additionally, these policies should encourage public awareness campaigns to educate visitors about the importance of IAQ to preserving both cultural heritage and public health.

Moreover, international cooperation is key in this realm. Sharing knowledge, research findings, and best practices between countries and institutions through international conferences, workshops, and collaborative research projects can lead to more effective and globally applicable solutions.

Finally, it is essential for these policies to be dynamic and adaptable. As new research emerges and technologies evolve, policies should be revisited and revised accordingly to ensure they remain effective and relevant. By taking a proactive and collaborative approach, policymakers, governments, and international organizations can significantly contribute to safeguarding the indoor air quality of historical buildings, thereby protecting both cultural heritage and the health of those who work in and visit these treasured sites.

In conclusion, optimizing the indoor air quality in historical buildings requires a comprehensive and multidisciplinary approach. By combining insights from environmental chemistry and environmental medicine with the latest developments in air purification and monitoring technologies, it is possible to create a safer and healthier environment for those who work in these culturally significant spaces. This not only protects the health of staff and visitors but also contributes to the preservation of these historical treasures for future generations.
